# Synergistic Effect of Repolarization of M2 to M1 Macrophages Induced by Iron Oxide Nanoparticles Combined with Lactate Oxidase

**DOI:** 10.3390/ijms222413346

**Published:** 2021-12-12

**Authors:** Zi-Xian Liao, Da-Liang Ou, Ming-Jung Hsieh, Chia-Chen Hsieh

**Affiliations:** 1 Institute of Medical Science and Technology, National Sun Yat-sen University, Kaohsiung 80424, Taiwan; m082060004@nsysu.edu.tw (M.-J.H.); whale51030@gmail.com (C.-C.H.); 2 Graduate Institute of Oncology, College of Medicine, National Taiwan University, Taipei 10051, Taiwan; dlou@ntu.edu.tw; 3 YongLin Institute of Health, National Taiwan University, Taipei 10051, Taiwan

**Keywords:** macrophage, iron oxide nanoparticles, lactate oxidase, lactate, synergistic effect

## Abstract

Metabolic reprogramming of tumors with the accompanying reprogramming of glucose metabolism and production of lactate accumulation is required for the subsequent development of tumors. Recent evidence has indicated that tumor-secreted lactate can promote an oncolytic immune microenvironment within the tumor. Furthermore, tumor-secreted lactate directly induces polarization of tumor-supportive M2 macrophages. However, oxidized tumor-secreted lactate in the tumor microenvironment can be exploited. Iron oxide nanoparticles have shown promising anticancer potential by activating tumor-suppressing macrophages. Furthermore, lactate oxidase (LOX) generally oxidizes tumor-secreted lactate and subsequently converts to pyruvate. Particularly, the ratio of M2 macrophages to M1 macrophages corresponds with tumor growth. In this study, we present iron oxide nanoparticles with carboxylic acid combined with LOX that enhance antitumor efficacy as a synergistic effect on the repolarization of tumor-supportive M2 macrophages to tumor-suppressive M1 macrophages in a tumor microenvironment. After M2 macrophages treated with iron oxide nanoparticles were combined with LOX, the ratio of M1 macrophages was significantly greater than iron oxide nanoparticles alone or with LOX alone. It is concluded that the inhibition of cancer cell proliferation by ratio of M1 macrophages was observed. This study suggests that the iron oxide nanoparticles combined with LOX could be potentially used for potentiating immune checkpoint inhibitor therapies for cancer treatment.

## 1. Introduction

The US Food and Drug Administration (FDA) has approved immune checkpoint inhibitors (ICIs), which show promise in changing the landscape of drug development for cancer therapy [1,2,3]. Furthermore, the efficacy of ICIs may be improved by enhancing antibody accumulation within targets [4] or generating synergistic immunomodulatory effects [5]. Tumor or cancer cells derive constituents to promote macrophages to a tumor-promoting status from a tumor-killing status. Immunotherapeutic approaches enhance T-cell-mediated immune responses and allow for control over polarization and recruitment of macrophages, especially to inhibit cancer progression [6,7]. Recent evidence has indicated that tumor-secreted lactate can promote an oncolytic immune microenvironment within the tumor [3,8]. Specifically, the extracellular level of lactate in the tumor microenvironment was shown to be higher than that under normal physiological conditions [8,9]. The tumor microenvironment typically consists of dynamically produced growth factors, cytokines, and metabolic product such as lactate. Thus, pH changes associated with lactate affect not only the immune system but also tumor growth [3,8,10,11]. However, tumor-secreted lactate plays a major role in processes other than the tumor microenvironment and immune cell functions, specifically polarization of tumor-supportive M2 macrophages [3].

**Figure 1:**
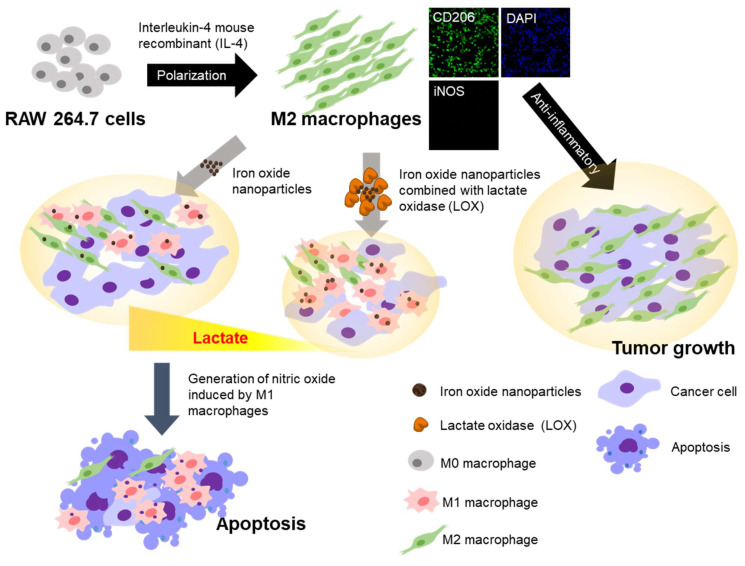
Concept of iron oxide nanoparticles combined with lactate oxidase (LOX) for synergistic effects on the distribution of macrophages. LOX specifically oxidized lactate in the tumor microenvironment, and the cellular uptake of iron oxide nanoparticles converted tumor-promoting M2 macrophages to tumor-suppressing M1 macrophages.

Nanomedicine was created from medical applications based on nanotechnology including nano-modified viruses/antibodies and biological nano-carriers [5,9,12,13,14,15]. Recently, iron oxide nanoparticles have attracted increasing attention in cancer immunotherapy [5,12,14,15]. Negatively charged iron oxide nanoparticles have exhibited promising anticancer potential by inducing macrophages from tumor-promoting M2 macrophages to tumor-suppressing M1 macrophages [12,15]. Due to the glycolytic tumor metabolism, the tumor microenvironment is usually acidic [3,8,9]. Lactate oxidase (LOX) enzymatically catalyzes lactate oxidation, producing pyruvate and H_2_O_2_. We previously developed a nanoparticle or hydrogel loaded with LOX [9,16] to provide specific targeting within lactate-rich tumor microenvironments. Furthermore, the in vitro or in vivo ratio of the M1/M2 macrophage corresponded with cancer cell proliferation or tumor growth [5,16]. Taken together, these results prompted the design of iron oxide nanoparticles with carboxylic acid combined with LOX for the repolarization of tumor-supportive M2 macrophages as reported here (Figure 1). We showed that consuming lactate and increasing cellular uptake of iron oxide nanoparticles can generate a synergistic effect on the repolarization of the M2 phenotype to the M1 phenotype and was associated with the inhibition of the proliferation of cancer cells. This approach was successfully verified with iron oxide nanoparticles combined with LOX at acidic pH conditions to induce lactate-mediated repolarization of macrophages and subsequently inhibited proliferation of cancer cells.

## 2. Results

### 2.1. Macrophages Incubated with Iron Oxide Nanoparticles Combined with LOX

**Figure 2:**
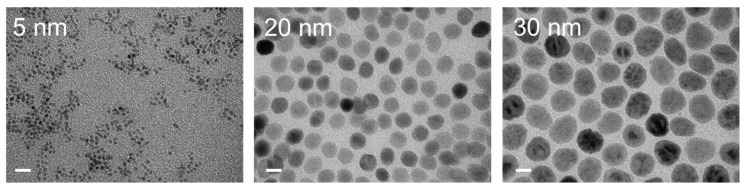
TEM images of various iron oxide nanoparticles with carboxylic acid. Bars = 20 nm.

The cytotoxicity of iron oxide nanoparticles (size: 5, 20, or 30 nm) only or iron oxide nanoparticles combined with LOX for a single dose or triple dose to M2 macrophages was determined by 3-(4,5-dimethylthiazol-2-yl)-5-(3-carboxymethoxyphenyl)-2-(4-sulfophenyl)-2H-tetrazolium (MTS) assays after culturing for 72 h. Transmission electron microscopy (TEM) photographs clearly revealed that the iron oxide nanoparticles had a diameter of approximately 5.0, 21.8, or 32.2 nm (Figure 2).

**Figure 3:**
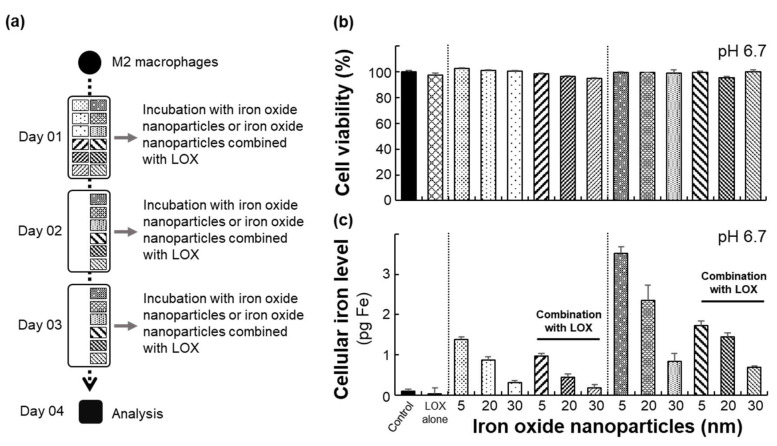
Characterization of materials: (**a**) Treatment protocol for M2 macrophages under various conditions. (**b**) Cell viability of macrophages after incubation with various treatments. Cell viability is given as the percentage of viable cells remaining after treatment for 72 h compared with that of the unexposed cells determined by the MTS assay. The bars represent the mean ± standard deviation (*n* = 4). (**c**) Qualitative determination of iron oxide nanoparticles in treated macrophages incubated in various conditions. The bars represent the mean ± standard deviation (*n* = 4).

**Figure 4:**
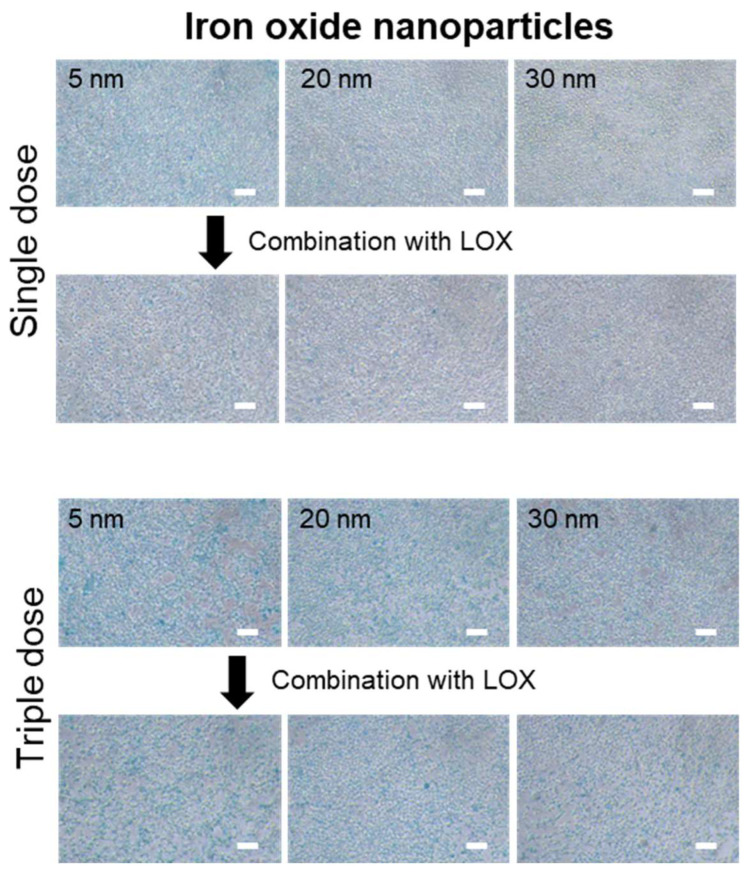
Cellular uptake of iron oxide nanoparticles. Representative images of macrophages after various treatments at day 3 with staining by an iron staining kit. Bars = 100 μm.

RAW 264.7 cells were given different treatments comprising single or triple doses over a period of 3 days according to the trials in Figure 3a. No significant toxicity was observed for the iron oxide nanoparticles alone or iron oxide nanoparticles combined with LOX incubated with M2 macrophages (Figure 3b). To measure the cellular uptake of iron oxide nanoparticles in M2 macrophages after treatment under different conditions, we quantified the treated M2 macrophages with an iron assay kit and observed the cells using an iron staining kit (Figure 3c). The cellular iron content significantly increased when M2 macrophages were incubated only with 5 nm iron oxide nanoparticles compared with iron oxide nanoparticles combined with LOX. Notably, the iron content decreased with iron oxide nanoparticles combined with LOX due to the aggregation (5 nm: 342 ± 93.6 nm; 20 nm: 783.4 ± 44.2 nm; 30 nm: 1165.6 ± 344.6 nm) induced by iron oxide nanoparticles and LOX. Consistent with the cellular iron content (Figure 3c), obvious cellular uptake of iron oxide nanoparticles was confirmed by staining with an iron staining kit (Figure 4).

### 2.2. Distribution of Macrophages with Various Treatments 

**Figure 5:**
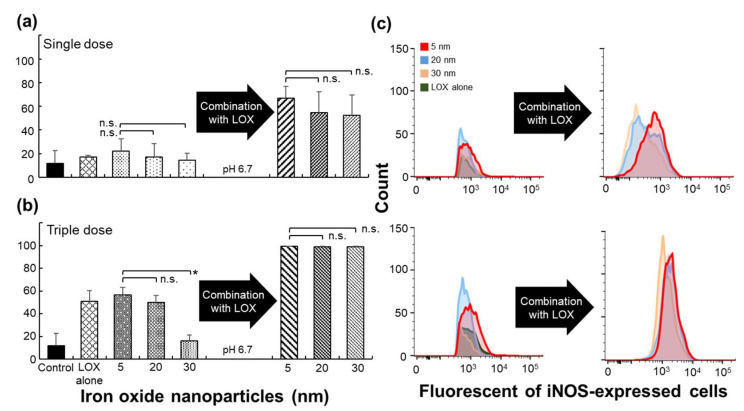
Repolarization of M2 to M1 macrophages. Percentages of M1 macrophages starting from M2 macrophages treated with various treatments at single doses (**a**) and triple doses (**b**) examined by flow cytometry (* *p* < 0.005; n.s., not significant, two-tailed unpaired Student’s *t*-test). Surface biomarkers, such as F4/80, iNOS, and CD206, were recognized macrophages of M1 or M2. The bars represent the mean ± standard deviation (*n* = 4). (**c**) Representative FACS results of (**a**,**b**).

**Figure 6:**
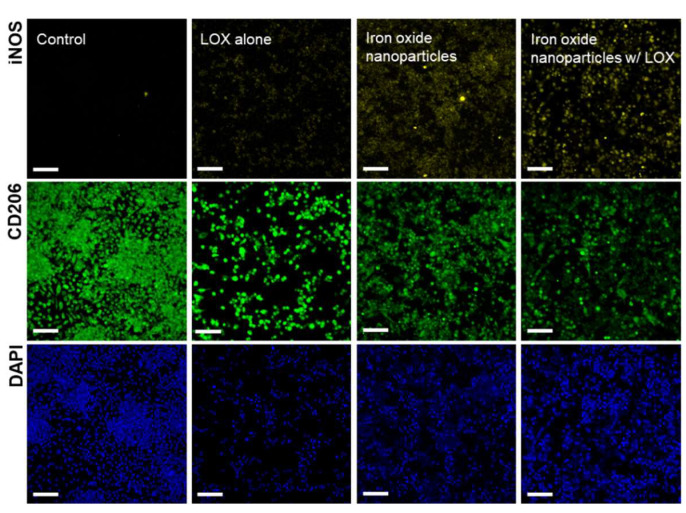
Distribution of macrophages under different treatments. Representative images of M1 macrophages incubated under various conditions. Bars = 100 μm.

Tumor-secreted lactate regulates immune function by polarizing M2 macrophages and promotes tumor growth [3,10]. We evaluated whether M2 macrophages can be repolarized to M1 macrophages when M2 macrophages exposed to LOX alone, iron oxide nanoparticles (size: 5, 20, or 30 nm), or iron oxide nanoparticles combined with LOX at a single dose or triple doses under an acidic pH after 3 days of incubation. When macrophages were incubated with a single dose or triple dose, the percentage of M1 macrophages stained with inducible nitric oxide synthase (iNOS) incubated with iron oxide nanoparticles combined with LOX was significantly greater (>3.0- or 2.0-fold) than that stained with iron oxide nanoparticles alone or LOX alone (Figure 5a–c). Notably, iron oxide nanoparticles combined with LOX had a synergistic effect on the repolarization of M2 to M1 macrophages, resulting in a significantly greater effect than that after the incubation of iron oxide nanoparticles or LOX. These results were confirmed by staining of F4/80^+^ iNOS^+^ CD206^−^ for M1 or F4/80^+^ iNOS^−^ CD206^+^s for M2 macrophages (Figure 6) and consistent with the flow cytometric analysis (Figure 5a–c). Taken together, these results reveal that lactate oxidizes into pyruvate by using LOX, thus decreasing the maintained M2 macrophages, and cellular uptake of iron oxide nanoparticles at the optimized particle size of 5 nm improves the repolarization of M2 to M1 macrophages.

### 2.3. Apoptosis Induced by M1 Macrophages

**Figure 7:**
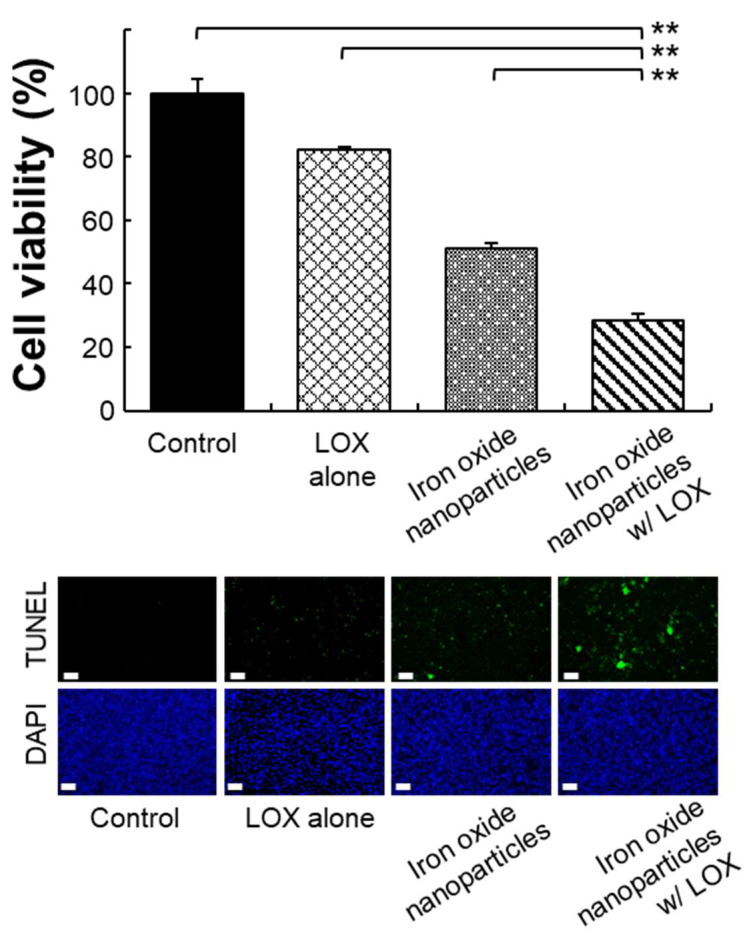
In vitro therapeutic effects. Cell viability of recombinant HT-1080 cells cocultured with M2 macrophages incubated with various treatments at pH 6.7 for 3 days as determined by MTS assays (** *p* < 0.0005, two-tailed unpaired Student’s *t*-test). The bars represent the mean ± standard deviation (*n* = 4). Microscopic images of apoptotic cells stained by TUNEL assay (green fluorescence). Bars = 100 μm.

Production of NO by M1 macrophages are characterized in bacterial endotoxin lipopolysaccharide (LPS) [16], and the total NO concentration was examined using the Griess assay. After M2 macrophages in acidic culture medium under iron oxide nanoparticles (5 nm) alone or iron oxide nanoparticles (5 nm) combined with LOX for a 3 day incubation period, treated macrophages were exposed to LPS (100 ng mL^−1^). From the results shown in Figure 5 and Figure 6, anticancer growth was performed on recombinant HT-1080 fibrosarcoma cells cocultured with M2 macrophages treated with iron oxide nanoparticles (5 nm) alone or iron oxide nanoparticles (5 nm) combined with LOX (Figure 7, upper panel). Specifically, HT-1080 cells exposed to M2 macrophage-incubated iron oxide nanoparticles (5 nm) combined with LOX at pH 6.7 significantly suppressed cancer cell growth (28.4%) compared to the HT-1080 cells exposed to M2 macrophages treated with iron oxide nanoparticles (51.2%) or LOX (82.2%) at pH 6.7. As expected, for HT-1080 cells treated with iron oxide nanoparticles (5 nm) combined with LOX, significant apoptosis signaling indicated by TUNEL (terminal deoxynucleotidyl transferase (TdT) dUTP nick-end labeling) assay was observed compared to iron oxide nanoparticles (5 nm) alone or LOX alone (Figure 7, lower panel). Taken together, we postulated that M2 macrophages were repolarized to M1 macrophages by treating with iron oxide nanoparticles and consuming lactate by LOX.

## 3. Discussion

Immunotherapy has shifted the paradigm for clinical cancer treatment; these therapies are purposed to improve antitumor immune responses [1,17,18]. In various immunotherapies, mediators are acted to induce or improve the stimulation of the immune system to attack cancer cells through natural mechanisms of immunosuppressive [1,17,18]. In clinical treatment, immunotherapy is considered a promising therapeutic strategy to treat and even cure several types of cancer. In general, cancer cells or solid tumors show increased levels of lactate relative to those in normal tissue, a phenomenon termed the Warburg effect [19]. For example, non-small cell lung cancer (NSCLC) is reprogramed the glucose metabolism and lactate production for activation of some kinases [3]. However, tumor-secreted lactate acts a key regulator for the modulation of immune systems in the tumor microenvironment [3].

**Figure 8:**
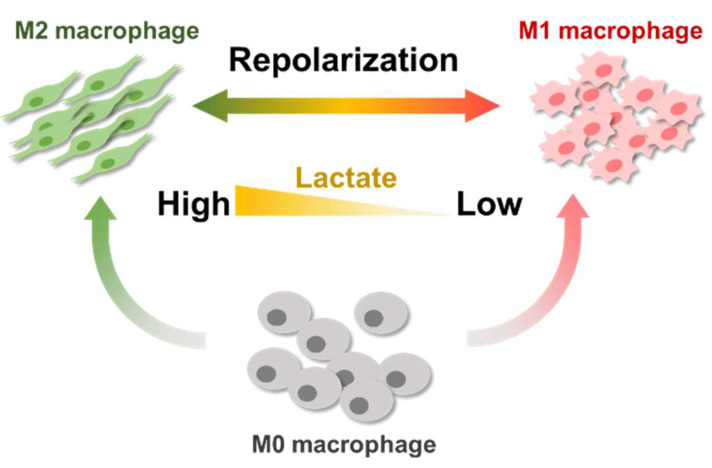
In vitro lactate directly modulates the distribution of M1/M2 macrophage from M0 macrophages.

Macrophages are mainly divided into two phenotypes: M1 (antitumoral, proinflammatory) and M2 (protumoral, anti-inflammatory). Specifically, tumor-secreted lactate can promote the suppressive functions of M2 macrophages and the subsequent tumor growth (Figure 8) [12,15,16]. Therefore, many studies have been associated with modulation of macrophages as an effective treatment to suppress tumor growth. Recently, extensive evidence has shown that tumor-secreted lactate depletion can improve current therapeutic strategies to restimulate immunosuppressive. Regarding lactate, inhibitors of lactate dehydrogenase A [20] or monocarboxylate transporters [21,22] have also been tested as a therapeutic strategy. However, the side effects associated with inhibition of lactate transport must be considered [21,22,23].

Current studies have demonstrated that LOX may target lactate in the tumor microenvironment; this enzyme can target lactate and is able to be combined with other therapeutic treatments [3,9,16,24,25]. Nanoparticles have changed the immunosuppressive environment in the tumor microenvironment by targeting the major components of the tumor microenvironment including macrophages, dendritic cells (DCs), and fibroblasts [5,12,14,15,26,27,28]. Additionally, iron oxide nanoparticles focus on anticancer immunotherapy by repolarizing macrophages from the M2 macrophage to the M1 macrophage. Furthermore, iron oxide nanoparticles specifically rely on the interferon regulatory factor 5 signaling pathway for polarization of M1 macrophages [15]. More interestingly, negatively charged iron oxide nanoparticles potently promote the induction of the M1 macrophages [29], and our results were consistently observed when M2 macrophages were treated with iron oxide nanoparticles with carboxylic acid of different sizes (5, 20, or 30 nm). In our results, 5 nm iron oxide nanoparticle uptake in M2 macrophages had a higher level (Figure 3c and Figure 4) compared with iron oxide nanoparticles of other sizes, leading to a significantly improved ratio of M1 macrophages (Figure 5 and Figure 6). More importantly, lactate was oxidized by LOX and subsequently converted to pyruvate. Consistent with our combined treatment, in a lactate-poor microenvironment, M2 macrophages can be repolarized and converted to M1 macrophages [16]. Overall, our study demonstrated that iron oxide nanoparticles combined with LOX generated a synergistic effect that reinvigorated the ratio of M1 macrophages and inhibition of cancer cells (Figure 7) as a potential anticancer therapy. Furthermore, mimicking the tumor microenvironment regulated RAW 264.7 cells as macrophages, and the obtained results will be verified by further in vivo experiments.

## 4. Materials and Methods

### 4.1. Materials

L-(+)-lactic acid, LOX from *Aerococcus viridians*, phosphate-buffered saline (PBS, pH 7.4), 4’,6-diamidino-2-phenylindole dihydrochloride (DAPI), an iron-staining assay, a nitrite/nitrate assay kit (colorimetric), and *Escherichia coli* O55:B5 LPS were purchased from Sigma–Aldrich Co. (St. Louis, MO, USA). The CellTiter 96^®^ AQueous One Solution Cell Proliferation Assay kit for the MTS assay was purchased from Promega (Madison, WI, USA). Recombinant mouse interleukin-4 (IL-4) was purchased from ProSpec-Tany TechnoGene, Ltd. (Rehovot, IL, USA). An iron assay kit (colorimetric) and anti-iNOS antibody were purchased from Abcam (Cambridge, MA, USA). Donkey anti-goat IgG (H+L) Alexa Fluor 488 and donkey anti-rabbit IgG (H+L) Alexa Fluor 555 were purchased from Invitrogen, Inc. (Carlsbad, CA, USA). Mouse MMR/CD206 antibody was purchased from R&D Systems (Minneapolis, MN, USA). The Apoptosis/Necrosis Detection Kit (green) was purchased from Abcam (Cambridge, UK).

Iron oxide nanoparticles (core size: 5, 20, and 30 nm) with carboxylic acid were purchased from Ocean NanoTech (San Diego, CA, USA). TEM (JEOL JEM-1400) was performed on a drop of iron oxide nanoparticles, which was air-dried onto a Formvar-carbon-coated 200 mesh copper grid. Images were acquired with an accelerating voltage of 100 kV.

### 4.2. Cell Culture and Polarization of M2 Macrophages 

Mouse RAW 264.7 (ATCC^®^ TIB-71™) M0 macrophages and human HT-1080 fibrosarcoma cells (ATCC^®^ CCL-121™) were cultured in Dulbecco’s modified Eagle’s medium (DMEM) with 10% fetal bovine serum (FBS), 3.7 g L^−1^ sodium bicarbonate, 100 μg mL^−1^ streptomycin, and 100 U mL^−1^ penicillin. Cells were maintained and cultured in a 37 °C incubator with 5% CO_2_.

For the polarization of M2 macrophages, M0 macrophages were incubated in culture medium containing 20 ng mL^−1^ IL-4 to generate M2 macrophages at pH 7.4 after 24 h of incubation [16]. Post-incubation, the macrophages were washed with PBS three times and used for M2 macrophages in subsequent studies at pH 6.7 adjusted by L-lactate (1.0 M).

### 4.3. Cytotoxicity of Materials

M2 macrophages (7 × 10^4^) were seeded in each of the wells of a 48-well plate and incubated in culture medium overnight. The M2 macrophages were then exposed to iron oxide nanoparticles (8.25 μg; 5, 20, or 30 nm) only or iron oxide nanoparticles with LOX (0.025 units) at a single dose or triple dose for 72 h of incubation. Macrophage proliferation was evaluated by CellTiter 96^®^ AQueous One Solution cell proliferation assay system, and the optical density (OD) of formazan at 490 nm was used to determine cell viability. The reagent contained a tetrazolium compound, 3-(4,5-dimethylthiazol-2-yl)-5-(3-carboxymethoxyphenyl)-2-(4-sulfophenyl)-2H-tetrazolium, and inner salt (MTS) and the reduction of MTS achieved by untreated cells was set at 100%, and that of test cells was expressed as a percentage of untreated cells [30,31]. Alternatively, the treated macrophages were quantized by an iron assay kit and observed using an iron-staining kit as described by the manufacturer.

### 4.4. Macrophages under Iron Oxide Nanoparticles Combined with LOX

M2 macrophages (7 × 10^4^) were seeded in each well of a 48-well plate and incubated in culture medium overnight. M2 macrophages were incubated with LOX (0.025 unit), 8.25 μg iron oxide nanoparticles (8.25 μg; 5, 20, or 30 nm) only or iron oxide nanoparticles with LOX (0.025 unit) at a single dose or triple dose at pH 6.7 for a 3 day incubation. Additionally, LPS induced the iNOS expression in M1 macrophages and led to increased pulmonary NO production [16]. The 9 × 10^4^ treated macrophages were cocultured with 3 × 10^4^ HT-1080 cells in each well of a 24-well plate and incubated in culture medium. Then, 100 ng mL^−1^ LPS was added to the culture medium for 24 h of incubation and evaluated by an in vitro assay or Apoptosis/Necrosis Detection Kit (green).

### 4.5. Macrophage Staining Assay

To evaluate the specificity of an M1 or M2 macrophage, we assessed surface markers such as iNOS and CD206. Macrophages were fixed with 4% paraformaldehyde (PFA), and immunostaining was performed using an anti-iNOS antibody and a mouse MMR/CD206 antibody specific to the markers iNOS and CD206 to observe the distribution of M1 or M2 macrophages. Immunofluorescence analysis of donkey anti-rabbit IgG (H+L) highly cross-adsorbed secondary antibody and Alexa Fluor 555 for iNOS or donkey anti-goat IgG (H+L) cross-adsorbed secondary antibody, Alexa Fluor 488 for CD206 was performed, and treated macrophages were observed with a confocal microscope and, subsequently, the nuclei of macrophages were stained with DAPI.

The immunostained macrophages were measured by flow cytometry (Beckman Coulter, Fullerton, CA, USA), and cells were appropriately gated by forward and side scatter, and 10,000 events per sample were collected. The negative control was used as untreated macrophages.

## 5. Conclusions

Macrophages are involved in mechanisms of immunosuppressive or anti-inflammatory in the tumor microenvironment. Our work based on iron oxide nanoparticles combined with LOX demonstrated that tumor-secreted lactate oxidation and cellular uptake of iron oxide nanoparticles repolarized protumor M2 macrophages to antitumor M1 macrophages. This study also suggests that iron oxide nanoparticles combined with LOX could generate a synergistic effect on regulating the distribution of macrophages in the tumor microenvironment with potential utility in potentiating ICI therapies.

## Data Availability

The data presented in this study are available upon request from the corresponding author.
